# Studying the mechanism and kinetics of fuel desulfurization using CexOy/NiOx piezo-catalysts as a new low-temperature method

**DOI:** 10.1038/s41598-023-34329-y

**Published:** 2023-05-10

**Authors:** Sangar S. Ahmed, Omid Amiri, Karwan M. Rahman, Savana J. Ismael, Noor S. Rasul, Darya Mohammad, Karukh A. Babakr, Nabaz A. Abdulrahman

**Affiliations:** 1 Chemistry Department, College of Science, Salahaddin University, Kirkuk Road, 44001 Erbil, Kurdistan Region Iraq; 2 Chemistry Department, College of Science, University of Raparin, Rania, Kurdistan Region Iraq; 3 Faculty of Chemistry, Razi University, Kermanshah, 67149 Iran; 4 Department of Petroleum and Mining Engineering, Faculty of Engineering, Tishk International University, Erbil, Iraq

**Keywords:** Environmental sciences, Natural hazards, Chemistry, Energy science and technology, Materials science, Nanoscience and technology

## Abstract

In order to advance desulfurization technology, a new method for excellent oxidative desulfurization of fuel at room temperature will be of paramount importance. As a novel desulfurization method, we developed piezo-catalysts that do not require adding any oxidants and can be performed at room temperature. A microwave method was used to prepare CeO_2_/Ce_2_O_3_/NiO_x_ nanocomposites. Model and real fuel desulfurization rates were examined as a function of synthesis parameters, such as microwave power and time, and operation conditions, such as pH and ultrasonic power. The results showed that CeO_2_/Ce_2_O_3_/NiO_x_ nanocomposites demonstrated outstanding piezo-desulfurization at room temperature for both model and real fuels. Furthermore, CeO_2_/Ce_2_O_3_/NiO_x_ nanocomposites exhibited remarkable reusability, maintaining 79% of their piezo-catalytic activity even after 17 repetitions for desulfurization of real fuel. An investigation of the mechanism of sulfur oxidation revealed that superoxide radicals and holes played a major role. Additionally, the kinetic study revealed that sulfur removal by piezo-catalyst follows a second-order reaction kinetic model.

## Introduction

Low-frequency vibration energy is everywhere in the environment and has a bright prospect for resolving the energy crisis and pollution issues. There has, however, been no effective utilization of this low-frequency energy^1,2^. Low-frequency vibration energy can be converted into energy in two ways: piezoelectric energy harvesting and piezoelectric catalysis^3–5^. As a result of piezoelectric catalysis, mechanical vibrations can be converted into free charges at the surface of piezoelectric materials in a wide range of environments (e.g. water and air). As a result of the local micro-electrolysis of water, piezo-catalytic materials can produce a number of reactive oxygen species (ROS) such as ·OH, ·O_2_^–^, ·HO_2_ and H_2_O_2_. In the textile, chemical, pharmaceutical, and food industries, ROS are used to catalytically oxidize and degrade toxic and carcinogenic dyes in water^6–8^.

The purpose of this work is to suggest a new application for piezocatalysts; we used nickel oxide/cerium oxide nanocomposites as piezocatalysts for the desulfurization of models and real fuels. Using Piezo-catalysis has several advantages compared to existing catalysis for desulfurization processes. They can operate in dark conditions, show a high desulfurization rate, and low cost, and can harvest low-frequency vibrations present in the environment to drive desulfurization reactions.

In spite of the adoption of new energy strategies in recent years, the use of conventional fuels remains dominant. As a result of the combustion of sulfur-containing fuels, SO_x_ is produced, which contributes to acid rain and fine particulate matter (PM 2.5), as well as corrosion of engines and catalytic converters^8–11^. Globally, strict regulations have been enacted to limit fuels with low sulfur content^12,13^. By utilizing hydrodesulfurization (HDS), which has been widely used in industry for decades, aliphatic sulfur can be easily removed from fuel oil in industrial production. Due to the steric hindrance, it is difficult to eliminate aromatic sulfur compounds from fuel, such as dibenzothiophene (DBTP). High temperatures and pressures, as well as large amounts of hydrogen, are required for HDS to remove thiophene derivatives, resulting in high operating costs and loss of octane^14–20^. We present here a piezocatalyst that eliminates sulfur compounds from the model and real fuel without adding any oxidants and by only applying mechanical force. In contrast, previous techniques for desulfurization required to add oxidants, such as H_2_O_2_^21–27^. Recently, an modified the oxidative desulfurization process by using single-atom catalysts with earth-abundant metal cores and robust nanoporous supports, as well as mixed transition metal oxides^28–30^. As a result of using single-atom catalysts or mixed transition metal oxides, the efficiency and stability of the catalyst were improved.

Different parameters, including synthesis parameters and operation parameters, were examined in order to determine how they affected the desulfurization rate. At room temperature, desulfurization rates of up to 92.7% for TP and 94.1% for DBTP could be achieved within 30 min of mechanical force is applied. The results showed that microwave pulses and calcination times had a dramatic impact on desulfurization rates in the synthesis step, whereas ultrasonic power and extraction time play a significant role in the operation step. In addition to its advantages, such as its oxidant-free nature and low reaction temperature, this method can be applied to basic, natural, and acidic environments.

## Experimental

### Material

There was no further purification of any of the chemicals used in the study since all of them were of analytical grade. Ce(NO_3_)_3_.6 H_2_O and Ni(NO_3_)_2_. 6 H_2_O were purchased from Fluka. Ethylenediaminetetraacetic acid disodium (EDTA-2Na) and ethanol were purchased from BDH Chemicals (England). 1, 4-benzoquinone (BQ), and Isopropanol (IPA) were purchased from Scharlau (Spain). Cetyltrimethylammonium Bromide (CTAB) and Ammonium hydroxide were purchased from Sigma-Aldrich and Merch, respectively.

### Synthesis and characterization of nickel oxide/cerium oxide nano composites Piezo catalyst

First, 30 mL of 0.06 mM Ce(NO_3_)_3_.6 H_2_O and Ni(NO_3_)_2_. 6 H_2_O was prepared, and the solvent was the mixture of distilled water and methanol with a ratio of 2:1, respectively. As a next step, the above solutions were mixed. An aqueous solution containing 0.016 mM CTAB was gradually added to the metal solution under stirring. By adding 1 M ammonia, the pH was adjusted to 11. Following this, the mixture was placed in a microwave and heated for four minutes (30 s on, 60 s off). The products were then washed, dried, and calcined at 500 °C for five hours. A series of catalysts were prepared under different parameters using the same procedure, as shown in Table 1. D1–D3 show the effect of microwave power, whereas d4 and d5 show the effect of microwave pulse. Lastly, d6–d11 illustrate the effects of microwave time, calcination temperature, and time.

**Table 1:** Experimental details for synthesis of CeO_2_/Ce_2_O_3_/NiO_x_ nanocomposites by microwave methods.

Catalyst	MW Power (W)	MW time (min)	MW pulse (on: off Sec)	Calcination temp, (℃)	Calcination time (h)
d1	600	4	30:60	500	5
d2	700	4	30:60	500	5
d3	900	4	30:60	500	5
d4	700	4	30:30	500	5
d5	700	4	30:10	500	5
d6	700	3	30:60	500	5
d7	700	6	30:60	500	5
d8	700	4	30:60	400	5
d9	700	4	30:60	700	5
d10	700	4	30:60	500	3
d11	700	4	30:60	500	7

### Desulfurization process by using piezo catalyst

In a Pyrex cell, 50 mg of piezocatalyst was used to oxidative desulfurize the model fuel and 25 mL of model fuel (TP or dibenzothiophene (DBTP)/n-hexane solution containing 500 ppm or real fuel (kerosene containing 2273 ppm) under the ultrasonic device. The samples were stirred in the dark for 30 min prior to ultrasonication. As a result of this step, equilibrium between adsorption and desorption is established. At certain intervals, desulfurized samples were withdrawn and centrifuged in a volume of 10 mL. The oxidized sulfur compounds were extracted with a 1:1 vol ratio of DMF under magnetic stirring for 15 min at room temperature after the precipitate was discarded. As a final step, two phases of DMF and hexane were separated and the model fuel in the upper phase was analyzed by an X-ray sulfur meter. The pH value of the solution was adjusted by adding drops of 0.1 M NaOH or 0.1 M H2SO4 in the pH range of 4–10 while stirring the solution. In order to measure the pH of solutions containing n-hexane solvent, a water extraction process is used. The sample is thoroughly mixed with water before being added (1:1). As soon as equilibrium has been reached, the solvent phase is separated, and the pH of the water phase is determined.

Based on the following formula, the desulfurization efficiency (DS) of fuel was calculated:

$$ {\text{DS}}\% \, = \,\left( {{\text{S}}_{0} - {\text{St}}/{\text{S}}_{0} } \right) \times {1}00. $$

During the initial and t-time periods, S and St represent the sulfur concentrations in the fuel solution, respectively.

In addition, radical trapping experiments were performed in order to identify the primary active species during piezo desulfurization. There were a number of scavengers added to the fuel system, including Na_2_-EDTA (to quench h +), IPA (to quench OH radical), and BQ (to quench ·O_2_^−^).

### Characterization techniques

An X-ray diffractometer (Philips X'pert Pro MPD, The Netherlands) was used to examine the crystal structure of the samples with Ni-filtered Cu Kα radiation (λ = 1.54 Å). Sonochemical reactions were carried out in an ultrasonic bath with a 20 kHz ultrasonic device with a maximum output power of 360 watts. An EDS (energy dispersion spectroscopy) analysis was performed with an X-Max Oxford, England. A FESEM image was recorded using IGMa/VP-ZEISS, Germany. Zeiss transmission electron microscopy was used to acquire TEM images.

## Results and discussions

### Characterizations of piezo-catalyst

**Figure 1:**
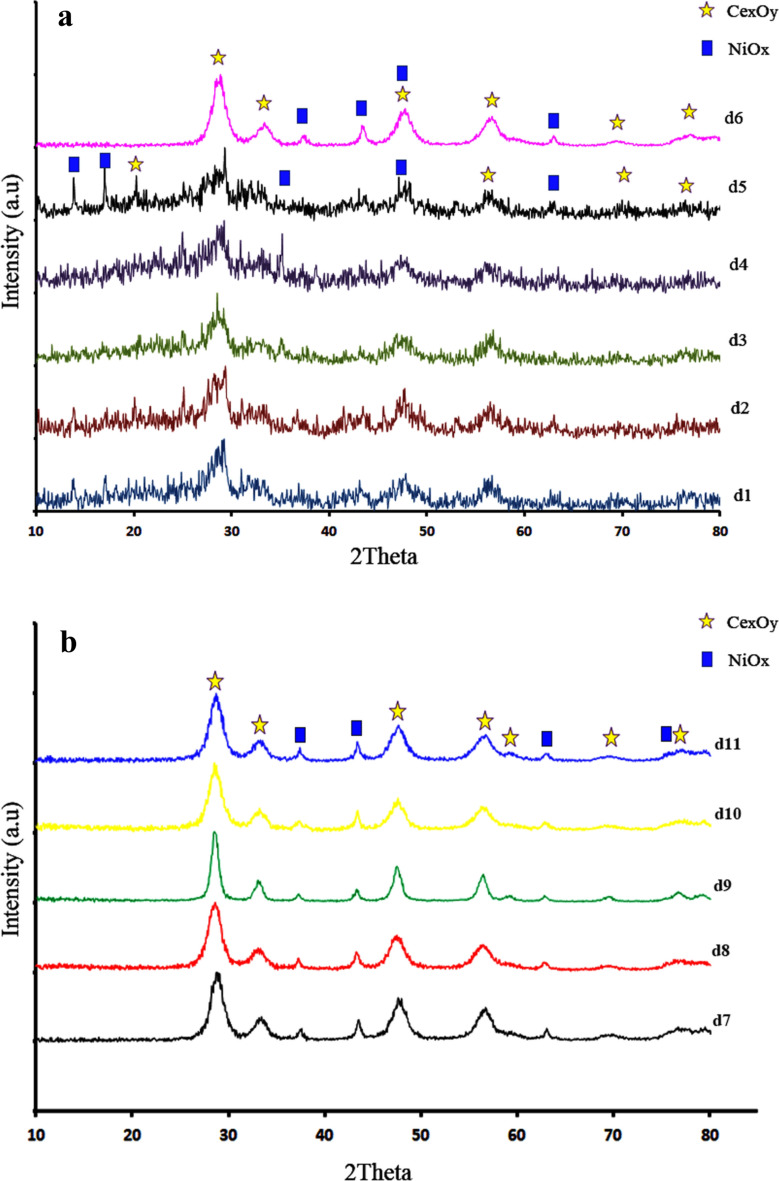
(**a**) XRD patterns of catalysts d1-d6 and (**b**) XRD patterns of catalyst d7-d11.

In order to determine the nature and phase structure of the as-synthesized samples, XRD analysis was conducted, as illustrated in Fig. 1. XRD pattern of d1-d3 indicated the effect of microwave power on crystallinity and compound of products. The CeO_2_/Ce_2_O_3_/NiO_x_ nanocomposite was formed under microwave power of 600 W. CeO_2_ and Ce_2_O_3_ showed good agreement with JCPDS 96-900-9009 with cubic phase and JCPDS 96-101-0280 with hexagonal phase. XRD patterns of nickel oxide with hexagonal phase were in good agreement with JCPDS 96-901-2317. Based on the Scherrer equation, an average crystal size of 17.9 nm was determined. A composite consisting of CeO_2_/Ce_2_O_3_/NiO_x_ with an average crystal size of 22.4 nm was obtained by increasing microwave power to 700 W. When microwave power is increased to 900 W (d3), however, CeO_2_ nanoparticles with an average crystal size of 4.9 nm are almost formed. Our previous research indicated that microwave pulses are an important parameter affecting the crystallinity and morphology of the final product^31–33^. The synthesis of catalysts was carried out under three different pulse conditions (30 s on, 60 s off, and 30 s on, 30 s off). An increase in the ratio of on to off in each cycle resulted in a decrease in the NiO_x_ ratio as shown in the XRD patterns. It appears that NiO_x_ requires more relaxation time in order to crystallize.

To investigate the effect of microwave time on the crystallinity and structure of final products, the catalyst was prepared at three different microwave times: 3 min (d6), 4 min (d2), and 6 min (d7). In accordance with the results of the XRD analysis, the intensity of the peak associated with nickel oxide decreased with increasing microwave time. Nickel oxide can be formed more efficiently by using less microwave energy. Calcination temperature and time are two other parameters in the synthesis of a catalyst. For the purpose of studying the effects of calcination temperature on the products, three different calcination temperatures were used: 400 °C (d8), 500 °C (d2), and 700 °C (d9). Based on our findings, Ce_2_O_3_ disappeared with increasing calcination temperature, leading to the formation of a composite composed of CeO_2_ and NiO. Increasing the calcination temperature also resulted in an increase in crystal size. The XRD patterns of d10, d2, and d11 illustrate the effect of calcination time on crystallinity. The results of this study showed that calcination time did not affect the composition of the composite. Ce_2_O_3_/CeO_2_/NiO_x_ nanocomposites were formed at three calcination temperatures of 3, 5 and 7 h. However, crystallinity improved with time.

**Figure 2:**
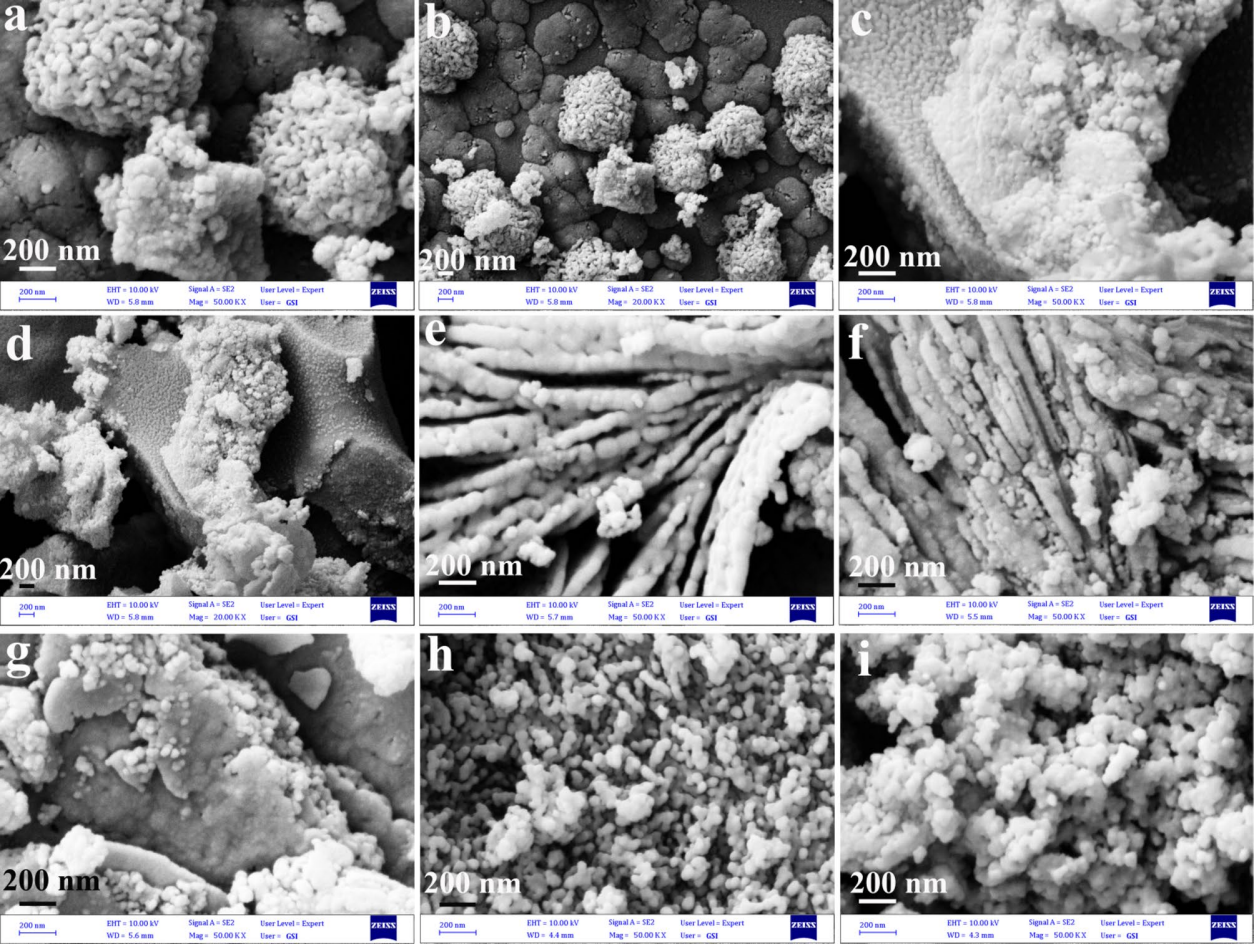
SEM images of : (**a,b**) catalyst d1, (**c,d**) catalyst d2, (**e**) catalyst d3, (**f**) catalyst d4, (**g**) catalyst d5, (**h**) catalyst d6, (**i**) catalyst d7.

An illustration of the SEM images of the d1-d7 catalysts is provided in Fig. 2a–i. The SEM images in Fig. 2a–e illustrate the effects of microwave power on the morphology of the products. The formation of micro-sized spheres made of particles 20–100 nm in diameter was observed using microwave power of 600 W (Fig. 2a and b). Increasing microwave power to 700 W led to the formation of micro-sized plates containing nanoparticles of 20–90 nm in diameter (Fig. 2c and d). The microwave power of 900 W resulted in rod-like structures made by nanoparticles with a diameter of 30–150 nm (Fig. 2e). In this study, it was demonstrated that microwave power can be used to control the morphology of a piezo-catalyst. Increasing microwave power resulted in changes in the morphology from microspheres to rods, as can be seen in the figure. Due to increasing power, the penetration depth of the solvent would increase, which would facilitate the formation of oriented structures like rods^34^.

**Figure 3:**
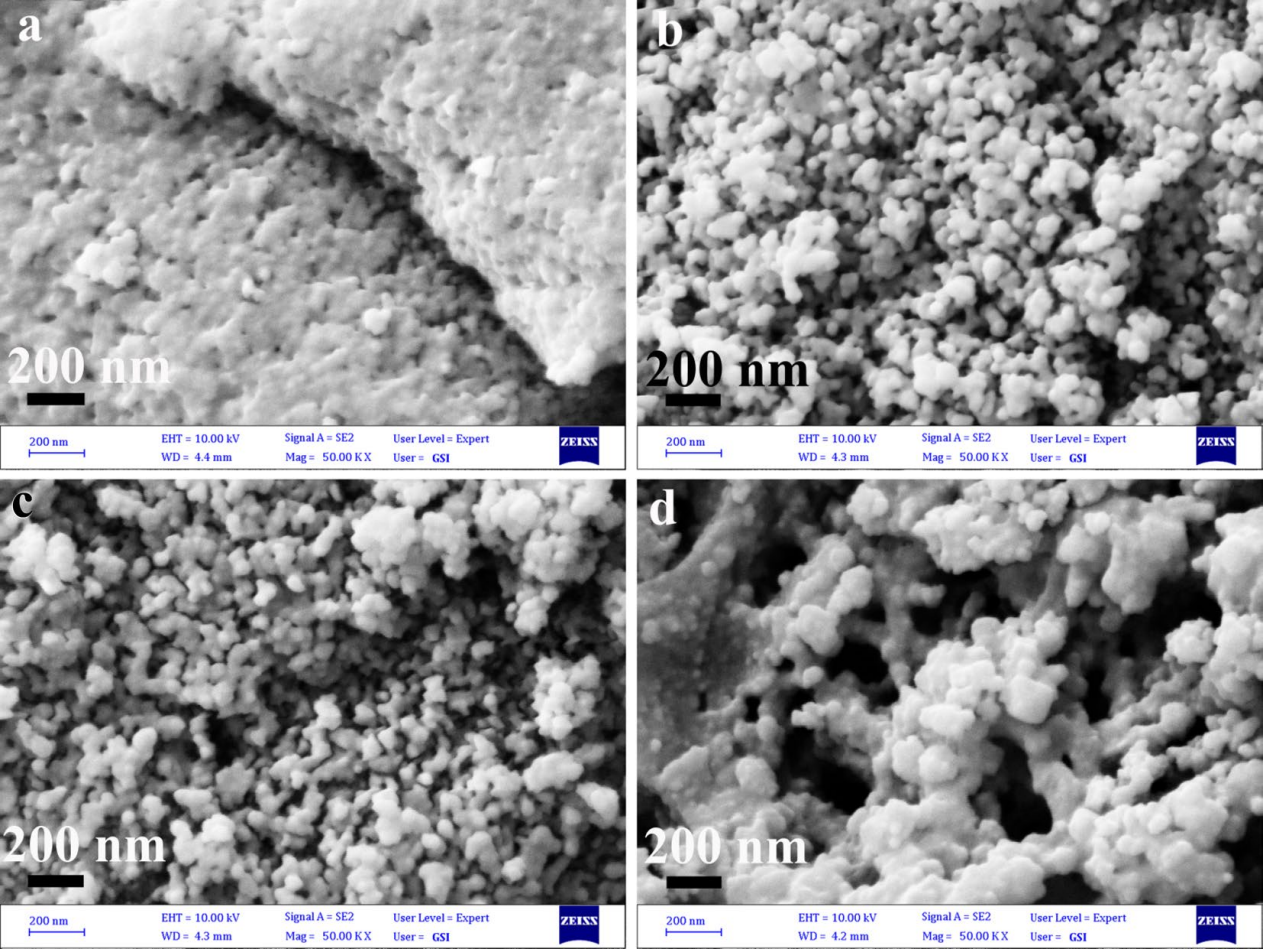
SEM images of: (**a**) catalyst d8, (**b**) catalyst d9, (**c**) catalyst d10, and (**d**) catalyst d11.

Changing the microwave pulse while keeping other parameters constant allowed a study of the effect of the microwave pulse on the morphology of piezo-catalysts. During the first series of products, microsphere structures were formed when the pulse was on for 30 s and off for 60 s (Fig. 2a and b). In Fig. 2f and Fig. S1 in SI, microwave pulses of 30 s on and 30 s off resulted in nanoplatelets with 40–120 nm in thickness that was adherent to each other. The thick plates with a thickness of 200–800 nm were formed when the pulse was 30 s on and 10 s off (Fig. 2g and Fig. S2 in SI). As a result of increasing the running time in each cycle, nanoparticles became more adherent. Increasing the running time in each cycle prevented nanoparticles from rearranging and assembling themselves. In addition, increasing the running time leads to a greater penetration depth, providing conditions for the formation of oriented structures^34^. Another parameter that might affect the morphology of the piezo-catalyst is microwave time. In Fig. 2a,b,h,i, SEM images demonstrate that nanoparticle sizes increased with increasing microwave time. In addition to calcination temperature, calcination time was another parameter that affected the morphology of final products. Nanoparticles with an average diameter of 18–80 nm were formed at a calcination temperature of 400 °C (Fig. 3a). It is shown in Figs. 2a and b that nanoparticles of 20–100 nm were obtained by increasing the calcination temperature to 500 °C. A subsequent increase in calcination temperature to 700 °C resulted in nanoparticles with a diameter of 30 to 150 nm (Fig. 3b). The results showed that nanoparticle size increased with increasing calcination temperature. To determine the effect of calcination time on the morphology of the piezo-catalyst, the piezo-catalyst was calcined for 3, 5, and 7 h. Nanoparticles with a diameter of 15–90 nm were formed after 3 h of calcination (Fig. 3c). During calcination time periods of 5 (Fig. 2a) and 7 h (Fig. 3d), piezo catalyst nanoparticles were enlarged to 20–100 nm and 50–200 nm, respectively.

**Figure 4:**
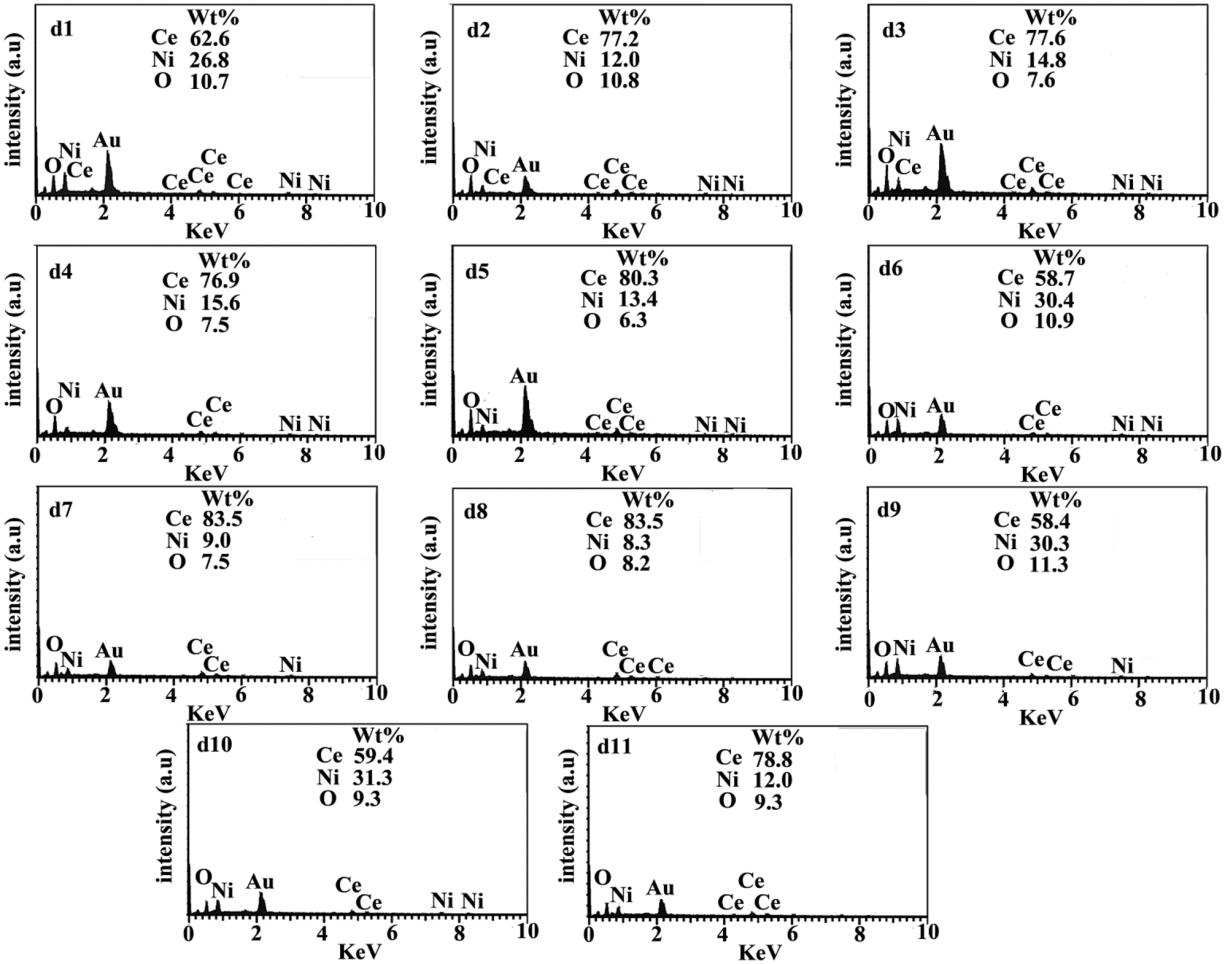
EDX results for catalyst d1-d11 with WT % for each element. Au was used to coat samples to prevent charge accumulation during taking EDX.

**Figure 5:**
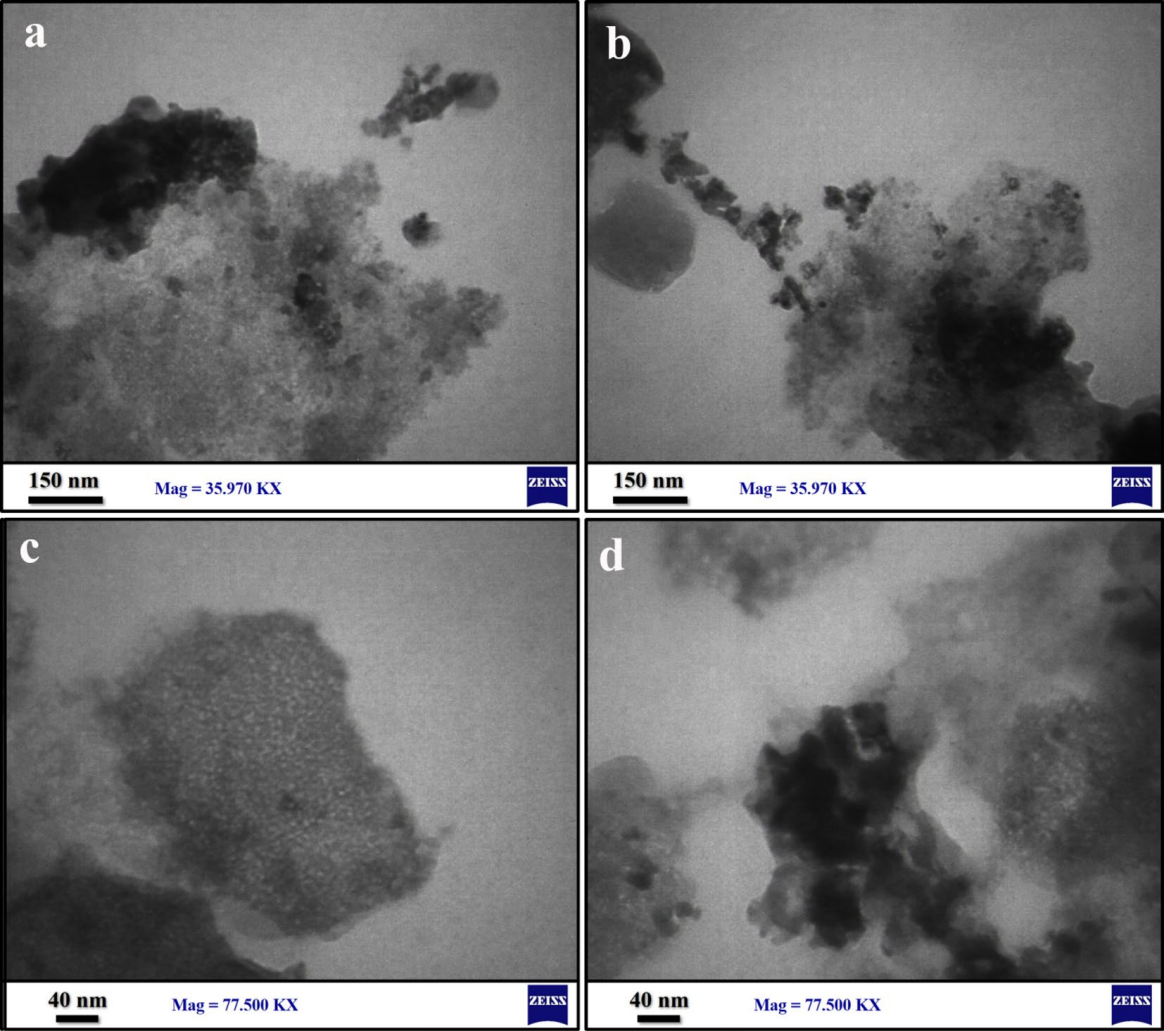
(**a–d**) shows the TEM images of d2 as the best piezo-catalyst in desulfurization of model and real fuel.

Figure 4 and Table 2 illustrate the effect of synthesis parameters and the ratio of Ce, Ce, and O in the piezo-catalysts. Based on EDX results shown in Fig. 4, peaks related to Ce, Ni, O, and Au have been detected, which confirms the purity of the final products. In order to improve the conductivity of samples and prevent charge accumulation during EDX measurements, Au was used. According to EDX results, increasing the microwave power from 600 W (d1) to 700 W (d2) increased the ratio of Ce, while decreasing the ratio of Ni, while maintaining an almost constant level of oxygen. When the microwave power is raised to 900 W (d3), the ratio of Ce remains nearly constant, whereas the ratio of Ni increases and the ratio of O decreases. As a result of comparing the EDS of d2, d4, and d5, we can conclude that increasing microwave running time in each cycle results in a higher Ce ratio while decreasing the O ratio. The Ce ratio in piezo-catalysts increased with an increase in microwave time from 3 min (d6) to 4 min (d2) and 6 min (d7). Increased calcination temperatures from 400 °C (d8) to 500 °C (d2) and 700 °C (d9) resulted in a decrease in Ce ratios and an increase in O ratios. As a result of the EDS of d10, d2, and d11, the element ratio in the piezocatalyst was affected by the calcination time. Extending the calcination time from 3 h (d10) to 5 h (d2) and 7 h (d10), the Ce ratio went up and the Ni ratio decreased. In Fig. 5a–d, TEM images of d2 are presented as a champion catalyst. On the basis of TEM results, d2 is almost uniform in diameter, ranging from 10 to 15 nm.

**Table 2:** EDX results, morphology and D% for samples d1-d11.

Catalyst	Ce ratio	Ni ratio	O ratio	Morphology	D % for TP	D % for DBTP
d1	62.6	26.8	10.7		81.7	82.4
d2	77.2	12.0	10.8		92.7	94.1
d3	77.6	14.8	7.6		87.4	86.9
d4	76.9	15.6	7.5		81.1	82.1
d5	80.3	13.4	6.3		72.4	74.6
d6	58.7	30.4	10.9		91.0	91.2
d7	83.5	9.0	7.5		89.3	89.2
d8	83.5	8.3	8.2		83.5	84.3
d9	58.4	30.3	11.3		91.7	92.6
d10	59.4	31.3	9.3		74.0	75.9
d11	78.8	12.0	9.3		91.5	92.0

### Effect of synthesis parameter on the piezo-desulfurization of fuel

**Figure 6:**
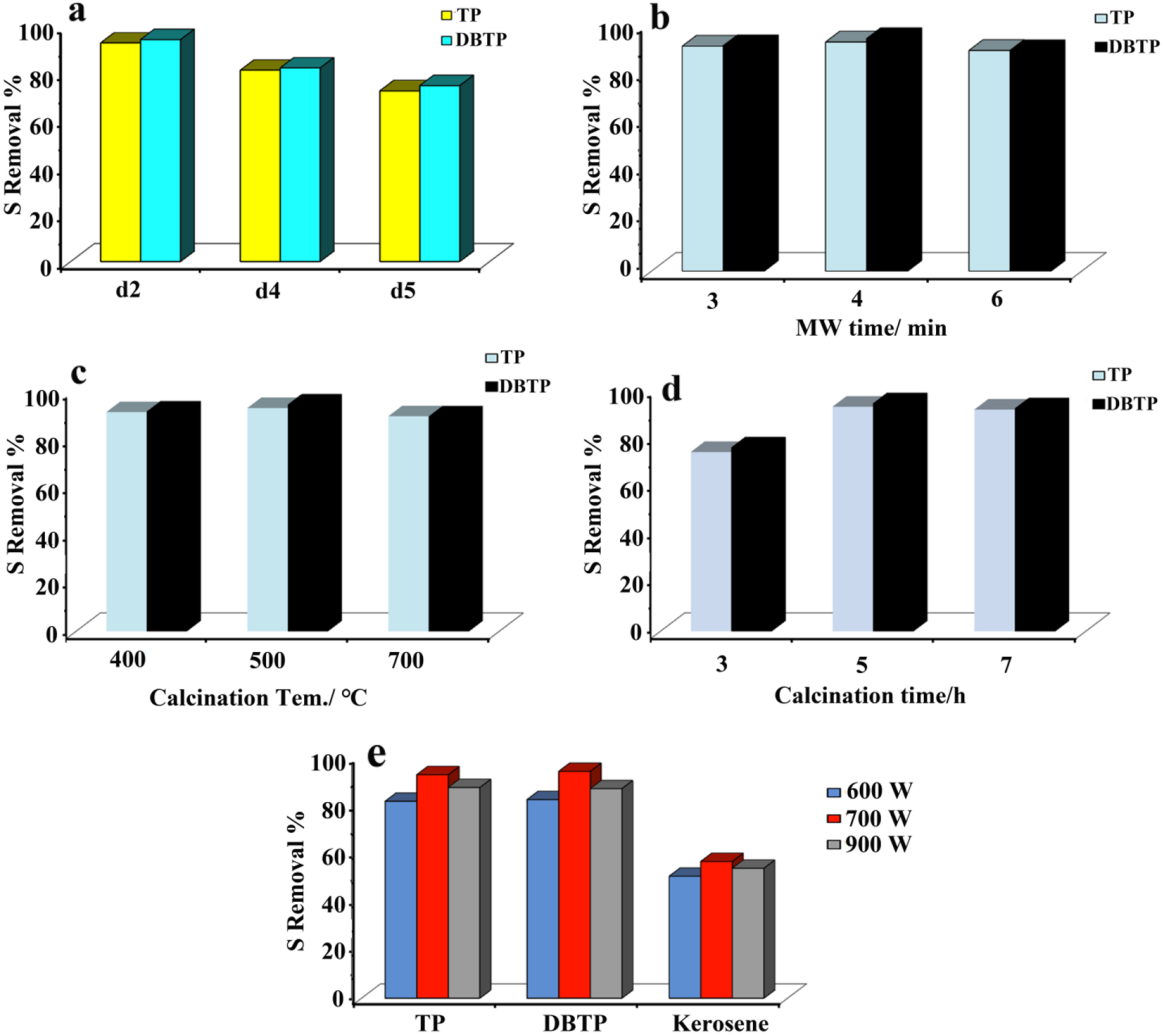
(**a**) Effect of microwave pulse during synthesis of catalyst on removal of TP and DBTP, (**b**) effect of microwave time during synthesis of catalyst on removal of TP and DBTP, (**c**) effect of calcination temperature during synthesis of catalyst on removal of TP and DBTP, (**d**) effect of calcination time during synthesis of catalyst on removal of TP and DBTP, (**e**) effect of microwave power during synthesis of catalyst on removal of TP, DBTP, kerosene.

Synthesis of parameters such as microwave power, microwave pulse, microwave time, calcination temperature, and time has a profound effect on desulfurization rats. In order to investigate these effects, piezo-desulfurization was carried out under ultrasonic power of 320 W for 30 min at pH = 7 and 50 mg of the catalyst. As can be seen in Fig. 6a, microwave pulses have a significant impact on the rate of desulfurization when piezo catalysts are prepared. Increasing microwave running time in each cycle significantly reduced desulfurization rates, as shown in the figure. The microwave running time during the synthesis of the catalyst was 30 s on and 60 s off in each cycle. This meant that 92.7 and 94.1% of the TP and DBTP were removed from the model fuel after only 30 min of ultrasonic processing. During the synthesis of the catalyst, the microwave pulse was changed to 30 s on and 30 s off. This resulted in a reduction in the desulfurization rate from 81.1 to 82.1% for TP and DBTP, respectively. In model fuel, TP and DBTP desulfurization rates were decreased to 72.4 and 74.6%, respectively, by changing the pulse duration to 30 s on and 10 s off during catalyst synthesis. The reason for this is that the d2 piezo catalyst has a smaller nanoparticle size compared to the d4 and d5. According to Kim et al.^35^, piezoelectricity increases non-linearly with decreasing nanostructure size.

Figure 6b illustrates the effect of microwave time during the synthesis of the catalyst on sulfur removal yield. According to the results, microwave time had no significant effect on desulfurization rates. As a result of using d6 (3-min microwaves) as a catalyst, TP and DBTP desulfurization rates were 91.0 and 91.2%, respectively. As a result of substituting the catalyst with d2, which was prepared by microwave irradiation for 4 min, 92.7 and 94.1% of TP and DBTP were removed from the fuel. Finally, when the catalyst prepared under 6 min microwave irradiation was used, the desulfurization rate for TP and DBTP was changed to 89.3 and 89.2% for TP and DBTP, respectively. As a result of the fact that microwave time did not significantly affect catalyst size, it did not appear to have a significant effect on desulfurization rates. For the same reason, the calcination temperature did not significantly affect desulfurization (Fig. 6c). The Piezo catalytic activity of products is influenced by the calcination time. As shown in Fig. 6d, the desulfurization rate is influenced by the calcination time during the synthesis stage. In the case of TP and DBTP, the removal rates were 74.0% and 75.9%, respectively, when the calcination time was 3 h (d10). As calcination time increased to 5 h (d2) and 7 h (d11), desulfurization rates increased to 92.7 and 91.5% for TP and 94.1 and 92.0% for DBTP, respectively. This could be attributed to the fact that the crystallinity of the catalyst increased with increasing calcination time. Last but not least, microwave power is considered to be an invaluable parameter in the synthesis of the catalyst that can influence the desulfurization rates of the model and real fuel (Fig. 6e). In Fig. 6e, it is shown that the optimum microwave power in desulfurizing TP, DBTP, and kerosene is 700 W. Using a piezo-catalyst synthesized at 600 W (d1) as the catalyst, 81.7, 82.4 and 50.6% of TP, DBTP in model fuel, and sulfur compounds in kerosene as real fuel were removed under 30 min ultrasonic treatment. When the catalyst was changed to d2, which was prepared at 700 W, desulfurization rates for TP, DBTP, and sulfur compounds in kerosene as real fuel increased by 92.7, 94.1 and 56.7%, respectively. Increasing the microwave power to 900 W, however, resulted in a decrease in the sulfur removal percentage to 87.4, 86.9 and 53.9%, respectively. In this case, smaller particles also demonstrated better piezo catalytic activity.

### Effect of operation condition on piezo removal of fuel

**Figure 7:**
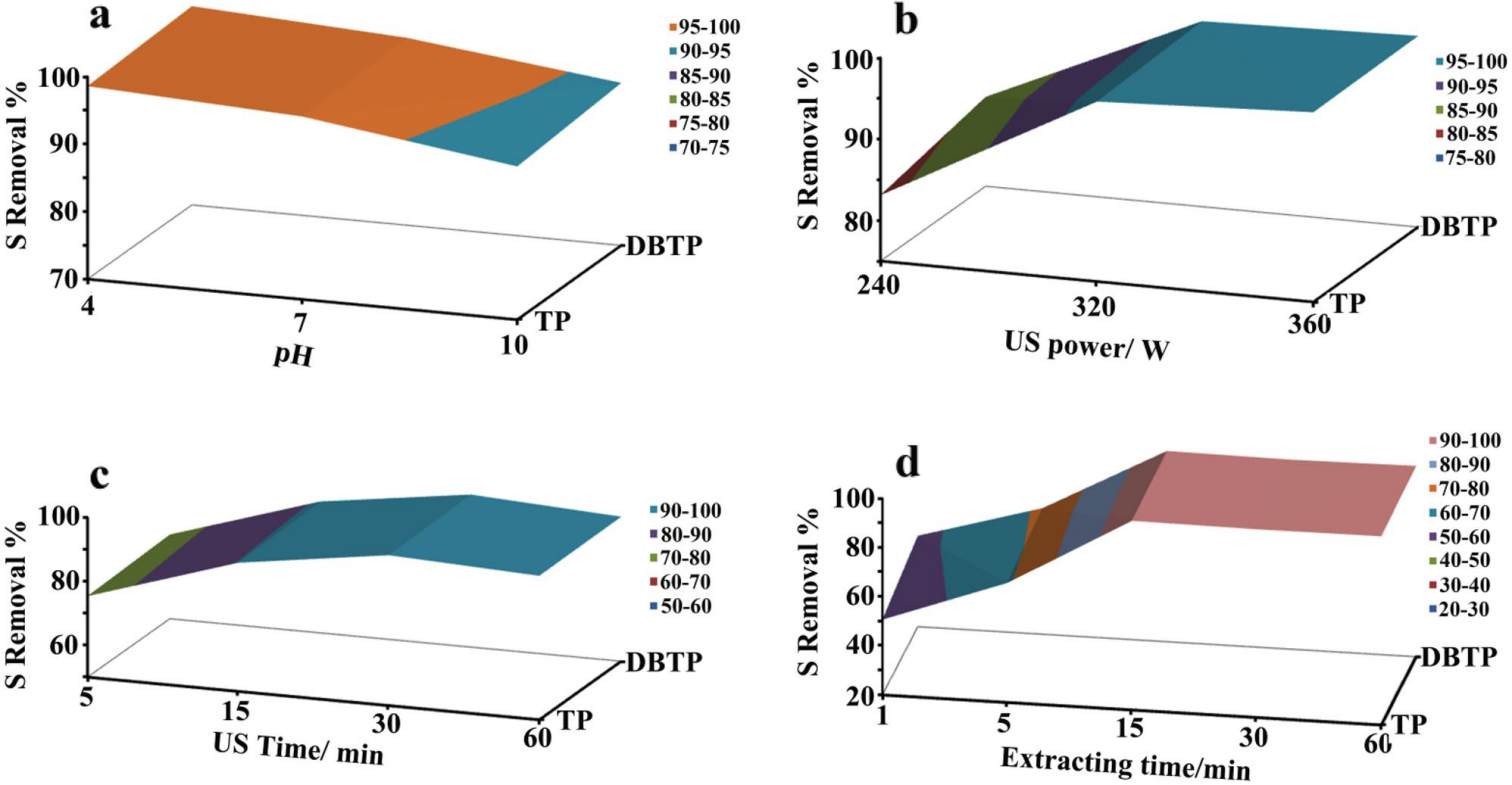
(**a**) Effect of pH of reaction in yield of piezo-desulfurization of TP and DBTP, (**b**) effect of ultrasonic power on desulfurization rate during piezo-desulfurization of TP and DBTP, (**c**) effect of ultrasonic time on desulfurization rate during piezo-desulfurization of TP and DBTP, (d) effect of extraction time on desulfurization rates of TP and DBTP.

As operation parameters that may influence desulfurization rates during piezo-desulfurization of fuel, we considered PH, ultrasonic power (US power), ultrasonic time (US time), and extracting time. Primary studies were conducted to identify optimum initial pH conditions for piezo-desulfurization of fuel. TP, DBTP, and sulfur compounds were desulfurized using d2 as the champion catalyst at pH 4, 7, and 10 (Fig. 7a and Table 3). During 30 min of ultrasonic treatment, 98.6 and 99.5% removal yields of TP and DBTP were achieved when the pH was initially adjusted to 4 using H_2_SO_4_. A pH of 7 resulted in a decrease in sulfur removal yields of 97.1 and 97.8% for TP and DBTP, respectively. When the pH was raised to 10 with NaOH, the desulfurization rate decreased even more. A pH adjustment to 10 resulted in the removal of 92.7 and 94.1% of TP and DBTP, respectively. Based on the results, it has been determined that acidic media are preferable for the piezo desulfurization of fuels. The reason for this is that H_2_SO_4_ can act as an oxidant and facilitate the oxidation of TP and DBTP. Alternatively, the basic environment decreases piezo-desulfurization since OH radicals played a negligible role in piezo-desulfurization and when we add more OH anions, more OH radicals are generated. We will discuss this in more detail in the section devoted to mechanisms. To test the effect of ultrasonic power on desulfurization rates, three power including 240 W, 320 W, and 360 W were used in piezo-desulfurization of TP and DBTP while pH was adjusted at 10 and 75 mg of d2 was used. Figure 7b and Table 3 showed that desulfurization rates increased by increasing ultrasonic power because piezo-electricity increased by increasing mechanical force and more free radical produced by increasing mechanical force.

**Table 3:** Details of piezo-desulfurization conditions and related desulfurization results for TP and DBTP.

Run	pH	US power (W)	US time (min)	Extraction time (min)	Catalyst dosage (mg)	TP/ DBTP content (ppm)	D% of TP	D% of DBTP
1	4	320	30	15	75	500	98.6	99.5
2	7	320	30	15	75	500	97.1	97.8
3	10	320	30	15	75	500	92.7	94.1
4	7	240	30	15	75	500	83.1	86
5	7	360	30	15	75	500	98.3	98.5
6	7	320	5	15	75	500	75.4	76.4
7	7	320	15	15	75	500	90.3	91.2
8	7	320	60	15	75	500	95.1	95.3
9	7	320	30	1	75	500	50.7	57.1
10	7	320	30	5	75	500	68.7	71.4
11	7	320	30	30	75	500	96.7	97.3
12	7	320	30	60	75	500	96.9	97.8
13	7	320	30	15	10	500	80.6	81.4
14	7	320	30	15	25	500	68.9	74.1
15	7	320	30	15	50	500	80.6	81.4
16	7	320	30	15	100	500	95.0	95.2
17	7	320	30	15	75	100	99.1	99.2
18	7	320	30	15	75	250	98.9	99
19	7	320	30	15	75	750	78.4	80.1
20	7	320	30	15	75	1000	64.2	63.4

Table 3 and Fig. 7c illustrate the effect of ultrasonic time. Results indicated that the removal of sulfur was increased by increasing ultrasonic time. After 5 min, approximately 75.4 and 76.44% of the TP and DBTP had been removed, indicating that the reaction was progressing at a rapid rate. The results showed that the reaction had almost reached equilibrium after 30 min.

**Figure 8:**
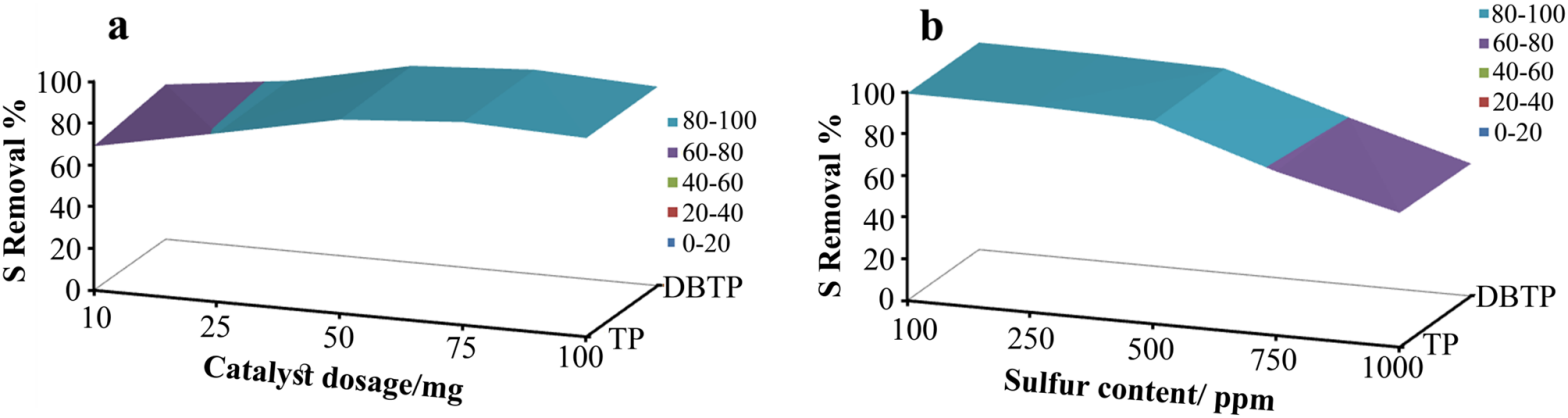
Effect of piezo-catalyst dosage (**a**) and sulfur content (**b**) on desulfurization rates of TP and DBTP.

During the desulfurization process, extracting time refers to the period during which oxidized sulfur compounds are extracted by a polar solvent while being shaken. A summary of the results is presented in Fig. 7d. The results indicated that extraction was completed within 15 min of shaking. When the extract time was 1 min, the desulfurization rate for TP and DBTP was 50.7 and 57.1%, respectively. As a result of increasing the extraction time to 5 min, the desulfurization rate for TP and DBTP increased to 68.7 and 71.4%, respectively. As a result of 15 min of extraction, the removal yield for TP and TBTP was 97.1 and 97.8%, respectively. After 15 min of extraction, desulfurization rates are almost constant, which is why we consider 15 min to be the optimal extraction time. The effects of catalyst dosage and sulfur content on desulfurization rates were demonstrated in Fig. 8 and Table 3. When the catalyst dosage was increased from 10 to 75 mg, removal yield increased, and then decreased slightly when the piezo-catalyst dosage was increased to 100 mg. When 75 mg catalyst was used to desulfurize model fuel, the removal yield was 97.1 and 97.8%, respectively. The removal yield decreased to 95.0 and 95.2% when a 100 mg catalyst was employed to desulfurize model fuel. As a result of high catalyst dosages, catalyst accumulation may occur which led to reduced desulfurization rates. When 10 ppm sulfur content was used in model fuel, 99.1 and 99.2% of TP and DBTP were eliminated. The sulfur content was increased to 250 ppm, resulting in the removal of 98.9 and 99.0% of TP and DBTP, respectively. A further increase in sulfur content to 500 ppm resulted in a slight decrease in the desulfurization rate, with the desulfurization rate for TP and DBTP changing to 97.1 and 97.8%, respectively. Furthermore, when sulfur content was increased to 750 ppm and 1000 ppm, desulfurization rates changed from 78.4 and 64.2% for TP to 80.1 and 63.4% for DBTP during 30 min ultrasonication.

### Reusability of piezo-catalyst in desulfurization of model and real fuel

**Figure 9:**
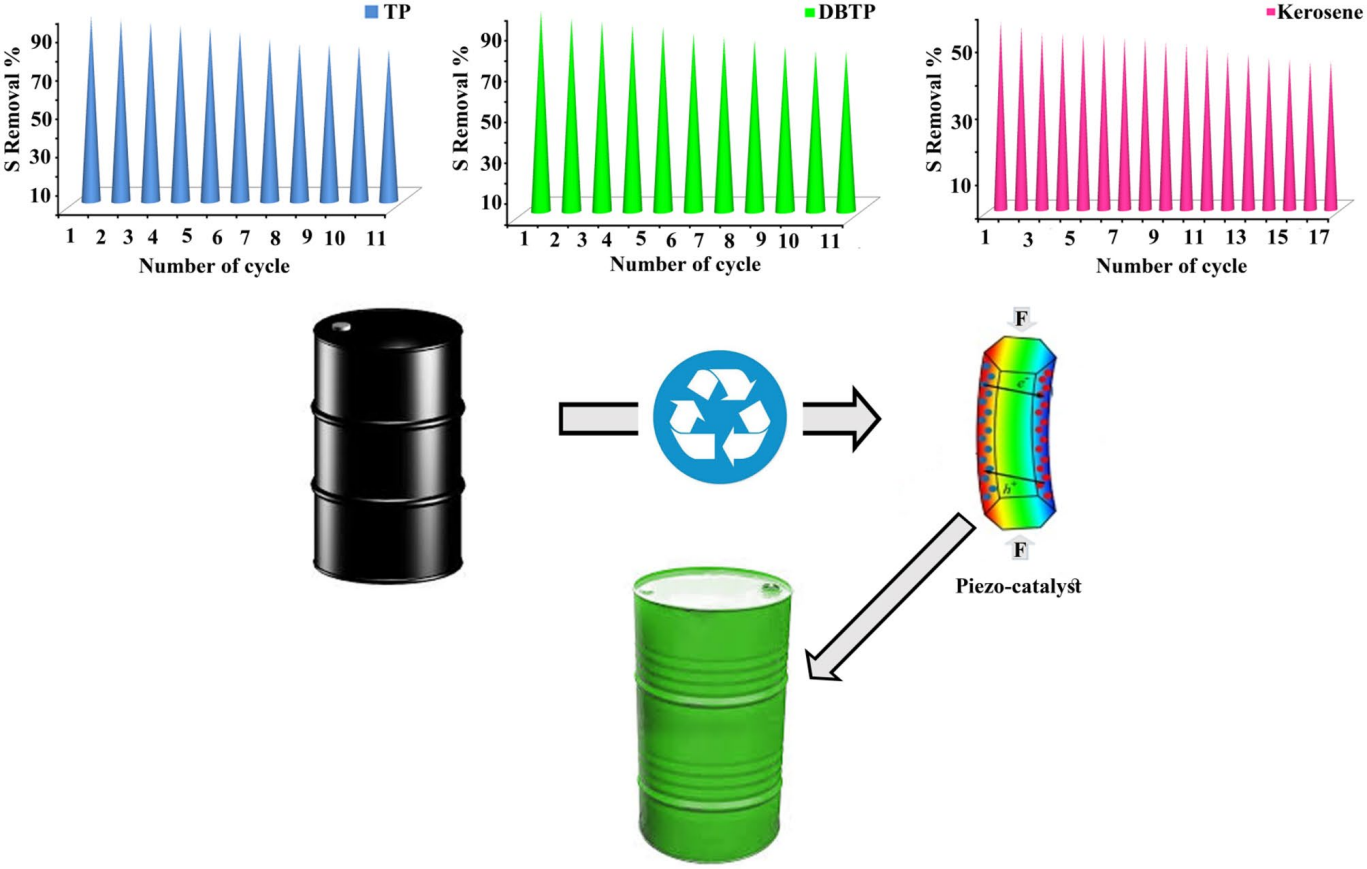
Reusability of piezo-catalyst in removing TP, DBTP and sulfur in kerosene. Catalyst was d2, catalyst dosage was 75 mg.

When considering the use of catalysts in large-scale industrial applications, recovering and reusing the catalyst is an imperative factor. We investigated the reusability of CeO_2_/Ce_2_O_3_/NiO_x_ piezo-catalyst, as shown in Fig. 9 and summarized in Table 4. Separating the used CeO_2_/Ce_2_O_3_/NiO_x_ piezo-catalyst from the reactant medium allowed it to be reused without prior treatment for the subsequent batch reaction. Reaction batches were conducted under the same conditions: reaction temperature of 30 °C, catalyst dosage of 75 mg, and pH value of 7. There was remarkable reusability of the piezocatalyst as determined by the results. In the first run, desulfurization rates for TP and DBTP were 97.1 and 97.8%, respectively. After 11 reuses of the catalyst, these rates decreased to 79.4 and 78.2%, respectively. Even after 11 times of re-use, it retained almost 82% of its piezo-catalytic activity. A prepared piezo-catalyst demonstrated even greater reusability when it was used to desulfurize real fuel (kerosene). Despite being run 17 times, it maintained almost 79% of its piezo-catalytic activity.

**Table 4:** Results for reusability of piezo-catalyst in desulfurization TP, DBTP, and kerosene.

Run no	Catalyst dosage (mg)	TP or DBTP content (ppm)	Kerosene sulfur content (ppm)	D % of TP	D % of DBTP	D % of kerosene
1	75	500	2273	97.1	97.8	56.7
2	75	500	2273	95.3	94.4	55.2
3	75	500	2273	93.5	92.9	53.2
4	75	500	2273	92.0	91.1	53.1
5	75	500	2273	91.0	90.3	52.8
6	75	500	2273	88.7	87.0	52.6
7	75	500	2273	84.9	85.3	51.9
8	75	500	2273	82.3	83.7	51.5
9	75	500	2273	82.2	80.6	50.6
10	75	500	2273	81.3	78.4	49.9
11	75	500	2273	79.4	78.2	49.6
12	75	–	2273	–	–	47.2
13	75	–	2273	–	–	46.8
14	75	–	2273	–	–	45.7
15	75	–	2273	–	–	45.3
16	75	–	2273	–	–	44.5
17	75	–	2273	–	–	44.7

### Mechanism of piezo-desulfurization of fuel

**Figure 10:**
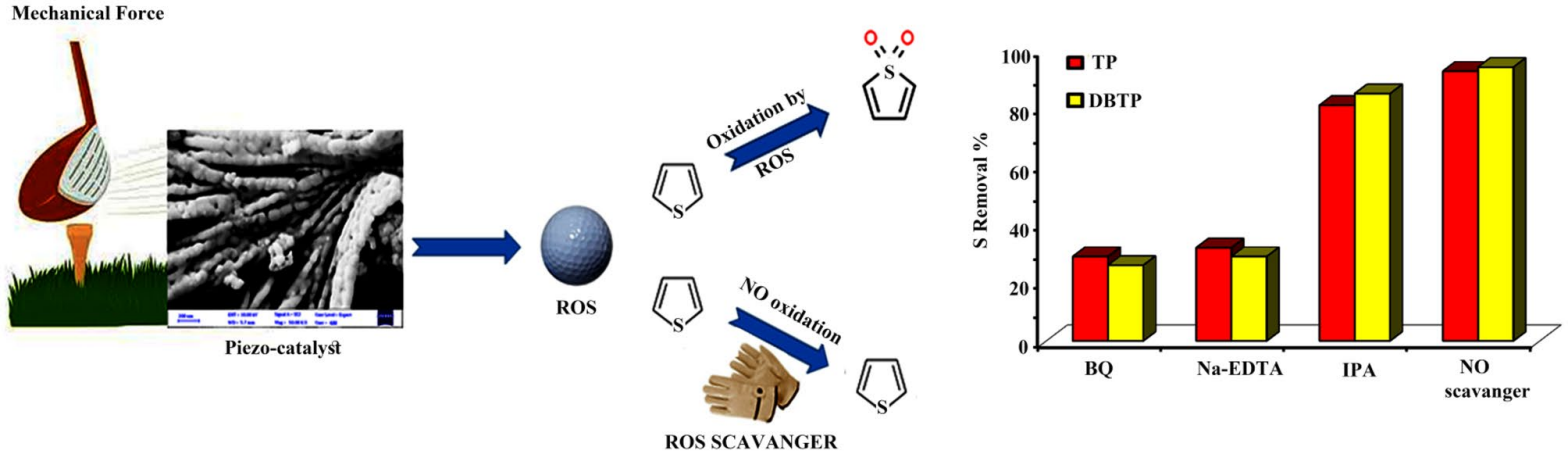
Schematic of piezo desulfurization of fuel and results for free radical scavengers.

To figure out the mechanism behind the piezo-desulfurization of catalysts, free radical scavengers experiments were conducted by using BQ, Na_2_EDTA, and IPA as superoxide radical scavengers, h + scavengers, and OH radical scavengers^5,36–38^. In the following batch reactions, the following conditions were used: reaction temperature of 30 degrees Celsius, d2 as a catalyst, piezo-catalyst dosage of 50 mg, and pH = 7. A summary of the results is shown in Fig. 10. Under 30 min of ultrasonic treatment, 92.7% and 94.1% of TP and DBTP were removed when no scavenger was used in the reaction. As a result of the addition of BQ, which acts as a superoxide radical scavenger, the removal percentage for TP and DBTP decreased to 29.0 and 26.0%, respectively. The results presented in Fig. 10 demonstrate that when Na_2_EDTA is used as an h + scavenger, the desulfurization rate for TP and DBTP decreases to 32 and 29.0%, respectively. In order to determine the effect of OH free radicals on fuel desulfurization, IPA was added to the reaction, which resulted in decreased desulfurization rates of 81.0 and 85.0%. A piezo desulfurization process was initiated by applying mechanical force to the piezo. The application of mechanical force resulted in the production of electrons and holes. Electrons react with trace oxygen in fuel and produce superoxide radicals, whereas holes react directly with sulfur compounds. A reaction between superoxide free radicals and holes led to the oxidation of sulfur compounds, which were then extracted with DMF. As a result of the results obtained, it was found that OH free radicals did not have a crucial role to play in the piezo-desulfurization of fuels. Due to the fact that the model fuel does not contain H_2_O, OH radicals played no significant role in the piezo desulfurization of TP and DBTP. TP and DBTP, on the other hand, were oxidized by holes and superoxide radicals generated by the piezo catalyst. It was approved by Jiang et al. that DBTO2 would form following the ODS process^29^. As a result of their feasible oxidation state, mixed transition metal oxides have been found to exhibit higher catalytic performances than single-component oxides. Moreover, binary metal oxides demonstrated higher catalytic performance, which may be attributed to the synergistic effect of the two metals^39^.

### Kinetic behind piezo-desulfurization of sulfur compounds by CeO_2_/Ce_2_O_3_/NiOx nanocomposite

**Figure 11:**
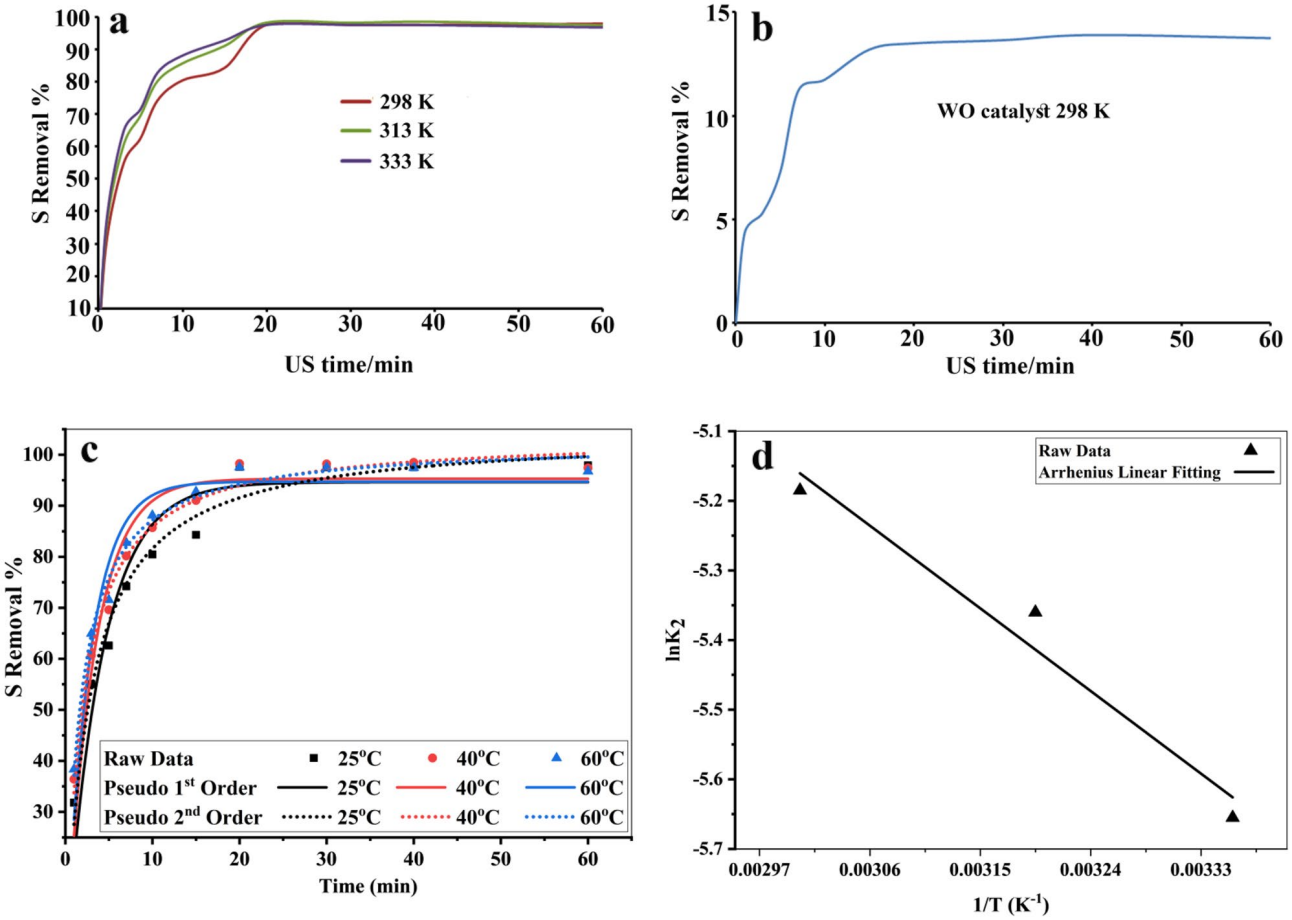
(**a**) piezo-desulfurization rates over time for 500 ppm TP in different temperatures in presence of d2 as catalyst, (**b**) piezo-desulfurization rates over time for 500 ppm TP without using catalyst, (**c**) Pseudo-first-order and pseudo-second-order fitting for piezo-removal of TP in different temperatures, (**d**) Activation energy for removal of TP by piezo catalyst based on Arrhenius equation.

A series of experiments were conducted in order to study the kinetics of piezo-desulfurization by CeO_2_/Ce_2_O_3_/NiO_x_ nanocomposite at different temperatures, including 298 K, 313 K, and 333 K. Figure 11a shows the rates of desulfurization at different temperatures for 500 ppm TP. As the reaction temperature was raised, the rate of sulfurization increased. The desulfurization rates for three different temperatures were close after 20 min of reactions and almost reached equilibrium after 20 min. Based on Fig. 11b, approximately 14.0% of the TP was removed after 60 min of ultrasonic treatment without a catalyst. There is evidence that CeO_2_/Ce_2_O_3_/NiO_x_ nanocomposite plays an influential role in desulfurization. The kinetics of piezo-desulfurization and characteristic constants of sulfur removal by piezocatalyst can be studied through kinetic measurements using pseudo-first-order, pseudo-second-order, and intra-particle diffusion. The Lagergren pseudo-first-order model is given as follows^26,40^:

1 $$ \frac{{{\text{dq}}}}{{{\text{dt}}}}\, = \,{\text{k}}_{{1}} \,\left( {{\text{q}}_{{\text{e}}} \,{-}\,{\text{q}}_{{\text{t}}} } \right). $$

The amounts of sulfur removed at equilibrium and at time t (min) are represented by qe and qt, respectively. k_1_ (min^−1^) is the rate constant.

Pseudo-second-order kinetic model assumes that the rate of degradation is second order^26,41,42^.

2 $$ \frac{{{\text{dq}}}}{{{\text{dt}}}}\, = \,{\text{k}}_{{2}} \left( {{\text{q}}_{{\text{e}}} {-}{\text{q}}_{{\text{t}}} } \right)^{{2}} $$

where k_2_ is the pseudo-second-order rate constant. According to nonlinear regression of the isotherm models, Fig. 11c and Table 5 show the kinetic parameters and their first and second order fitting at different temperatures (298 K, 313 K, and 333 K). It was estimated that Pseudo-First-Order fitting at 298 K had an R^2^ of 0.927, whereas Pseudo-Second-Order fitting had an R^2^ of 0.978. In the case of piezo-desulfurization of fuel at 313 K, R^2^ for pseudo-first and pseudo-second orders changed to 0.934 and 0.987, respectively. At 333 K, the R^2^ for pseudo-first order and pseudo-second order was 0.935 and 0.987, respectively. Clearly, the results indicated that the desulfurization process was governed by a second order kinetics for CeO_2_/Ce_2_O_3_/NiO_x_ nanocomposite piezo-catalyst. This explains why the reaction rate is so high in the first ten minutes. The activation energy was 10.968 kJ/mol based on the liner Arrhenius equation for second order rate constant desulfurization of sulfur compound by CeO_2_/Ce_2_O_3_/NiO_x_ nanocomposites (Fig. 11d).

**Table 5:** Kinetic Parameters for piezo-desulfurization of TP in different temperatures.

Kinetic models	Parameters	Temperature (K)		
		298	313	333
Pseudo-first-order	*q*_*e*_ (Removal %)	94.6	95.3	94.7
	*K*_*1*_ (min^−1^)	0.243	0.309	0.357
	*R*^*2*^	0.927	0.934	0.935
Pseudo-second-order	*q*_*e*_ (mg. g^−1^)	104.3	103.7	102.5
	*K*_*2*_ (g. mg^−1^. min^−1^)	0.0035	0.0047	0.0056
	*R*^*2*^	0.978	0.987	0.987

Activation energy for second order rate constant in non-linear fitting is (10.412 kJ. mole^−1^).

Activation energy for second order rate constant in linear fitting is (10.968 kJ. mole^−1^).

## Conclusion

We have developed a new method for desulfurizing real and model fuel by the use of CeO_2_/Ce_2_O_3_/NiOx nanocomposites with PIEZO-catalytic properties. Desulfurization was achieved here without adding any oxidant and at room temperature. The XRD, EDS, EDS, and TEM characterizations confirmed that the CeO_2_/Ce_2_O_3_/NiO_x_ nanocomposites were successfully synthesized. In addition to its outstanding desulfurization activity, the PIEZO-catalyst achieves complete desulfurization within 20 min at room temperature without adding any oxidants. It was possible to remove up to 97.1 and 97.8% of 500 ppm of TP and DBTP, respectively, in 30 min without adding any oxidant. CeO_2_/Ce_2_O_3_/NiO_x_ nanocomposite showed excellent reusability, maintaining 79% of its piezo-catalytic activity even after 17 cycles. In addition, the effect of synthesis parameters such as microwave time, microwave power, calcination temperature, and calcination time, and operation parameters such as pH, reaction temperature, ultrasonic power and time on sulfur compounds removal was examined. As a result of studying the mechanism behind the piezo-desulfurization reaction, it was found that superoxide radicals and holes played the primary roles in the oxidation of sulfur compounds. As well, the process of desulfurization by CeO_2_/Ce_2_O_3_/NiO_x_ nanocomposites follows a second order reaction.

## Supplementary Information

Supplementary Figures.

## Data Availability

All data generated or analyzed during this study are included in this published article (and its Supplementary Information files).

## References

[1] Lim XB, Ong WJ (2021). A current overview of the oxidative desulfurization of fuels utilizing heat and solar light: From materials design to catalysis for clean energy. Nanoscale Horiz..

[2] Lu HY, Li PC, Deng CL, Ren WZ, Wang SN, Liu P, Zhang H (2015). Deep catalytic oxidative desulfurization (ODS) of dibenzothiophene (DBT) with oxalate-based deep eutectic solvents (DESs). Chem. Commun..

[3] Tang NF, Zhang YN, Lin F, Lu HY, Jiang ZX, Li C (2012). Oxidation of dibenzothiophene catalyzed by [C8H17N(CH3)3]3H3V10O28 using molecular oxygen as oxidant. Chem. Commun..

[4] Chowraymond J, Kopp J, Portney PR (2003). Energy resources and global development. Science.

[5] Guan Sh, Li Zh, Xu B, Wu J, Wang N, Zhang J, Han J, Guan T, Wang J, Li K (2022). Cyclodextrin-based deep eutectic solvents for efficient extractive and oxidative desulfurization under room temperature. Chem. Eng. J..

[6] Balmaceda MM (2018). Differentiation, materiality, and power: Towards a political economy of fossil fuels. Energy Res. Soc. Sci..

[7] Hao J, Gong Zh, Yang Zh, Tian Y, Wang Ch (2022). Sulfur types distribution and the fast thermal-cracking behaviors of sulfur-containing compounds in Buton oil sand bitumen. Fuel.

[8] Zhou X, Wang T, Zhang L, Che Sh, Liu H, Liu S, Wang Ch, Su D, Teng Zh (2022). Highly efficient Ag2O/Na-g-C3N4 heterojunction for photocatalytic desulfurization of thiophene in fuel under ambient air conditions. Appl. Catal. B.

[9] Haruna A, Aljunid Merican ZM, Musa SG, Abubakar S (2022). Sulfur removal technologies from fuel oil for safe and sustainable environment. Fuel.

[10] Wang H, Jibrin I, Zeng X (2020). Catalytic oxidative desulfurization of gasoline using phosphotungstic acid supported on MWW zeolite. Front. Chem. Sci. Eng..

[11] Kulkarni PS, Afonso CAM (2010). Deep desulfurization of diesel fuel using ionic liquids: Current status and future challenges. Green. Chem..

[12] Chen S, Zhao C, Liu Q, Zang M, Liu C, Zhang Y (2018). Thermophilic biodesulfurization and its application in oil desulfurization. Appl. Microbiol. Biotechnol..

[13] Becker CM, Marder M, Junges E, Konrad O (2022). Technologies for biogas desulfurization—An overview of recent studies. Renew. Sustain. Energy Rev..

[14] Seredych M, Bandosz TJ (2011). Removal of dibenzothiophenes from model diesel fuel on sulfur rich activated carbons. Appl. Catal. B.

[15] Zhang P, Xu Y, Guo K, Yin Y, Wang J, Zeng Y (2021). Hierarchical-pore UiO-66 modified with Ag+ for π-complexation adsorption desulfurization. J. Hazard. Mater..

[16] Xu J, Zhang B, Lu Y, Wang L, Tao W, Teng X, Ning W, Zhang Z (2022). Adsorption desulfurization performance of PdO/SiO2@graphene oxide hybrid aerogel: Influence of graphene oxide. J. Hazard. Mater..

[17] He J, Wu Y, Wu P, Lu L, Deng C, Ji H, He M, Zhu W, Li H (2020). Synergistic catalysis of the PtCu alloy on ultrathin BN nanosheets for accelerated oxidative desulfurization. ACS Sustain. Chem. Eng..

[18] Shafiq I, Shafique S, Akhter P, Ishaq M, Yang W, Hussain M (2021). Recent breakthroughs in deep aerobic oxidative desulfurization of petroleum refinery products. J. Clean. Prod..

[19] Zou J, Lin Y, Wu Sh, Wu M, Yang Ch (2021). Construction of bifunctional 3-D ordered mesoporous catalyst for oxidative desulfurization. Sep. Purif. Technol..

[20] Zhou X, Wang T, Liu H, Gao X, Wang C, Wang G (2021). Desulfurization through photocatalytic oxidation: A critical review. Chemsuschem.

[21] Shafiq I, Hussain M, Shafique S, Rashid R, Akhter P, Ahmed A, Jeon JK, Park YK (2021). Oxidative desulfurization of refinery diesel pool fractions using LaVO4 photocatalyst. J. Indus. Eng. Chem..

[22] Morshedy AS, Ali HR, Nad AA, Rabie AM, El-Maghrabi HH (2021). Highly efficient imprinted polymer nanocomposites for photocatalytic desulfurization of real diesel fuel. Environ. Technol. Innov..

[23] Amiri O, Salar Kh, Othman P, Rasul T, Faiq D, Saadat M (2020). Purification of wastewater by the piezo-catalyst effect of PbTiO3 nanostructures under ultrasonic vibration. J. Hazard. Mater..

[24] Amiri O, Abdalrahman A, Jangi G, Ahmed HA, Hussein SH, Joshaghani M, Mawlood RZ, Salavati-Niasari M (2022). Convert mechanical energy to chemical energy to effectively remove organic pollutants by using PTO catalyst. Sep. Purif. Technol..

[25] Ismael SJ, Amiri O, Ahmed SS (2022). Preparation and characterization of La1-xMn1-yO3 piezocatalyst for removing waste drug pollutants in wastewater under the piezo-catalyst effect. Sep. Purif. Technol..

[26] Babakr KA, Amiri O, Guo LJ, Rashi MA, Mahmood PH (2022). Kinetic and thermodynamic study in piezo degradation of methylene blue by SbSI/Sb2S3 nanocomposites stimulated by Zirconium Oxide balls. Sci. Rep..

[27] Liu Zh, Zhao K, Xing G, Zheng W, Tang Y (2021). One-step synthesis of unique thorn-like BaTiO3–TiO2 composite nanofibers to enhance piezo-photocatalysis performance. Ceram. Int..

[28] Jiang W, An X, Xiao J, Yang Zh, Liu J, Chen H, Li H, Zhu W, Li H, Dai Sh (2022). Enhanced oxygen activation achieved by robust single chromium atom-derived catalysts in aerobic oxidative desulfurization. ACS Catal..

[29] Jiang W, Xiao J, Gao X, An X, Leng Y, Zhu L, Zhu W, Li H (2021). In situ fabrication of hollow silica confined defective molybdenum oxide for enhanced catalytic oxidative desulfurization of diesel fuels. Fuel.

[30] Jiang W, Zhu K, Li H, Zhu L, Hua M, Xiao J, Wang Ch, Yang Zh, Chen G, Zhu W, Li H, Dai S (2020). Synergistic effect of dual Brønsted acidic deep eutectic solvents for oxidative desulfurization of diesel fuel. Chem. Eng. J..

[31] Wen Y, Chen J, Gao X, Che H, Wang P, Liub B, Ao Y (2022). Piezo-enhanced photocatalytic performance of ZnO nanorod array for pollutants degradation in dynamic water: Insight into the effect of velocity and inner flow field. Nano Energy.

[32] Gu J, Ahna J, Jung J, Cho S, Choi J, Jeong Y, Park J, Hwang S, Cho I, Ko J, Ha JH, Zhao ZJ, Jeon S, Ryu S, Jeong JH, Park I (2021). Self-powered strain sensor based on the piezo-transmittance of a mechanical metamaterial. Nano Energy.

[33] Joo H, Lee KY, Lee J-H (2022). Piezo/triboelectric effect driven self-powered gas sensor for environmental sensor networks. Energy Technol..

[34] Sugimoto H, Biggemann J, Fey T, Singh P, Khare D, Dubey AK, Kakimoto K (2021). Lead-free piezoelectric (Ba, Ca)(Ti, Zr)O3 scaffolds for enhanced antibacterial property. Mater. Lett..

[35] Deka S, Devi MB, Khan MR, Venimadhav KA (2022). Piezo-photocatalytic and photocatalytic bismuth vanadate nanorods with antibacterial property. ACS Appl. Nano Mater..

[36] Ahn CW, Song HC, Nahm S, Park SH, Uchino K, Priya S, Lee HG, Kang NK (2005). Effect of MnO2 on the piezoelectric properties of (1–x)(Na0.5K0.5)NbO3–xBaTiO3 ceramics. Jpn. J. Appl. Phys..

[37] Park H-Y, Nam Ch-H, Seo I-T, Choi J-H, Nahm S, Lee H-G, Kim K-J, Jeong S-M (2010). Effect of MnO2 on the piezoelectric properties of the 0.75 Pb(Zr0.47Ti0.53)O3–0.25Pb(Zn1/3Nb2/3)O3 ceramics. J. Am. Ceram. Soc..

[38] Guo J, Chu L, Yang H, Huang Zh, Yang M, Wang G (2023). Amphiphilic halloysite nanotube enclosing molybdenum oxide as nanoreactor for efficient desulfurization of model fuels. Chem. Eng. J..

[39] An X, Zhu L, Xiao J, Jiang W, Gao X, Xu L, Li H, Zhu W, Li H (2022). Engineering hollow mesoporous silica supported cobalt molybdate catalyst by dissolution-regrowth strategy for efficiently aerobic oxidative desulfurization. Fuel.

[40] Ning Zh, Jiang Y, Jian J, Guo J, Cheng J, Cheng H, Chen J (2020). Achieving both large piezoelectric constant and high Curie temperature in BiFeO3-PbTiO3-BaTiO3 solid solution. J. Eur. Ceram. Soc..

[41] Yang F, Zhu X, Wu J, Wang R, Ge T (2022). Kinetics and mechanism analysis of CO2 adsorption on LiX@ZIF-8 with core shell structure. Powder Technol..

[42] Wang H, Shen H, Shen C, Li Y, Ying Zh, Duan Y (2019). Kinetics and mechanism study of mercury adsorption by activated carbon in wet oxy-fuel conditions. Energy Fuels.

