# Supplementary material for: Studying the mechanism and kinetics of fuel desulfurization using CexOy/NiOx piezo-catalysts as a new low-temperature method
Source: Sci Rep. 2023 May 10;13:7574. doi: 10.1038/s41598-023-34329-y (PMC10172175; doi:10.1038/s41598-023-34329-y)
Supplement: Supplementary file 1 — Supplementary Figures. [file 41598_2023_34329_MOESM1_ESM.docx]

**Studying the mechanism and kinetics of fuel desulfurization using CexOy/NiOx piezo-catalysts as a new low-temperature method**

*Sangar S. Ahmed ^a^,* *Omid Amiri ^b, c *^, Karwan M. Rahman ^a^, Savana J. Ismael ^a^, Noor S. Rasul ^a^, Darya Mohammad ^a^, Karukh A. Babakr ^b^, Nabaz A. Abdulrahman ^d^*

*^a^ Chemistry Department, College of Science, Salahaddin University, Kirkuk Road, 44001, Erbil, Kurdistan Region, Iraq*

*^b^ Chemistry Department, College of Science, University of Raparin, Rania, Kurdistan Region, Iraq*

*^c^* *Faculty of Chemistry, Razi University, Kermanshah 67149, Iran*

*^d^ Department of Petroleum and Mining Engineering, Faculty of Engineering, Tishk International University, Erbil, Iraq*

* Corresponding author. Tel: +9647700581175

E-mail address: [o.amiri1@gmail.com](mailto:o.amiri1@gmail.com), [oamiri@uor.edu.krd](mailto:oamiri@uor.edu.krd)

* Corresponding author. Tel: +9647700581175

E-mail address: [o.amiri1@gmail.com](mailto:o.amiri1@gmail.com), [oamiri@uor.edu.krd](mailto:oamiri@uor.edu.krd)


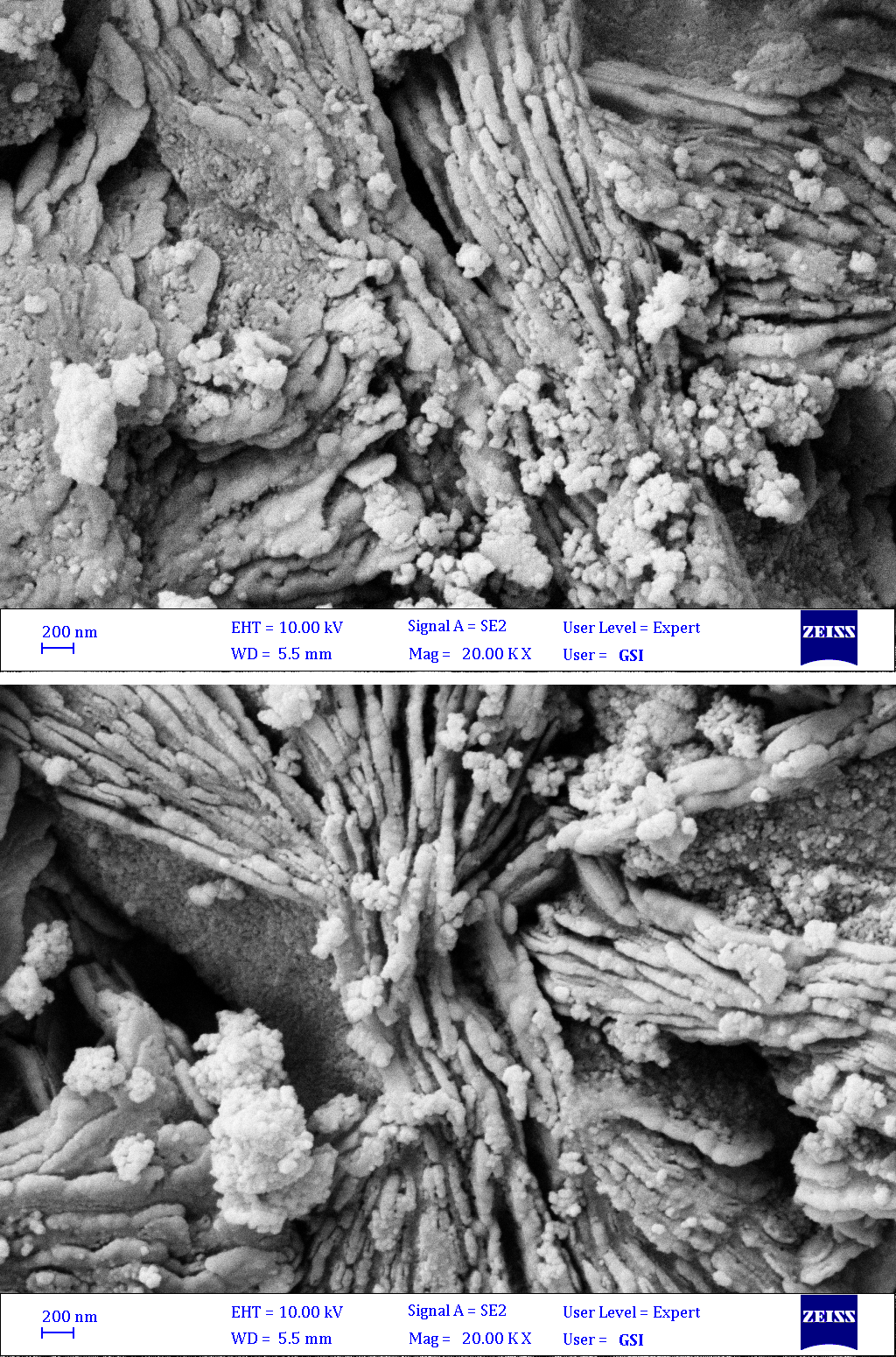


**Figure S1**. SEM images of sample prepared when pulse was on for 30 seconds and off for 30 seconds. These images indicated how plates stuck together.


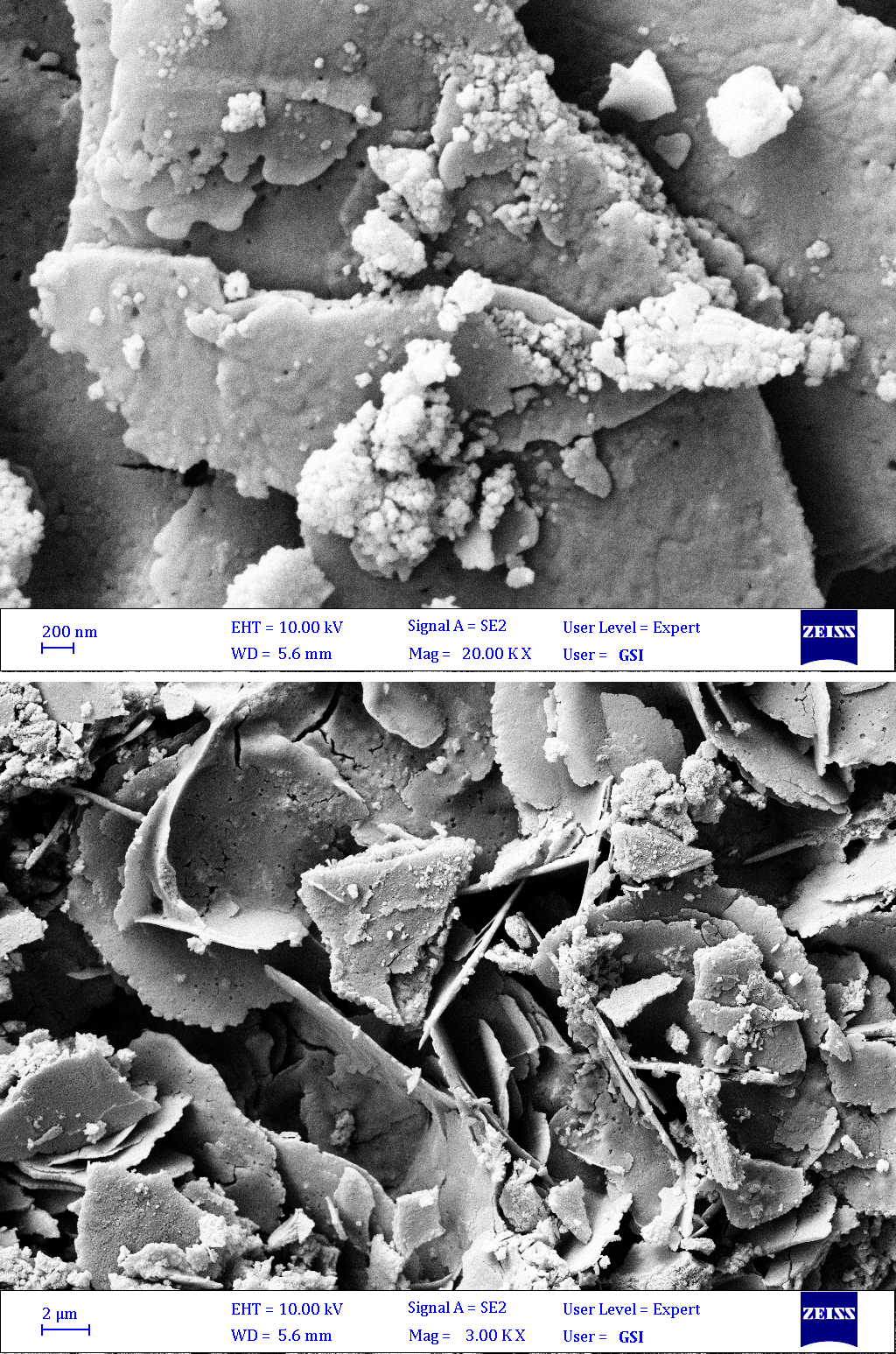


**Figure S2**. SEM images of sample prepared when pulse was on for 30 seconds and off for 10 seconds. These images indicated how plates stuck together.
